# Intracoronary microcatheter indwelling for continuous pumping of low-dose thrombolytic drugs: A new approach to reperfusion in acute myocardial infarction

**DOI:** 10.1097/MD.0000000000037692

**Published:** 2024-04-05

**Authors:** Yuzhu Chen, Yuan Liu, Xiaohui Li, Guozhen Jin

**Affiliations:** a Department of Cardiology, Hongze District People’s Hospital, Huaian, China; b Department of General Practice, Huaihai Road Community Health Service Center, Nanjing, China; c Department of Radiology, Children’s Hospital, Nanjing Medical University, Nanjing, China; d Department of Cardiology, Nanjing First Hospital, Nanjing Medical University, Nanjing, China.

**Keywords:** acute myocardial infarction, coronary thrombolysis, reperfusion therapy, thrombolytic therapy

## Abstract

Reperfusion therapy of acute myocardial infarction (AMI) refers to physical or chemical recanalization and restoration of blood flow to an occluded coronary artery, and current techniques for reperfusion therapy include intravenous thrombolysis, percutaneous coronary intervention (PCI) and coronary artery bypass grafting (CABG). The number of patients receiving emergency CABG in the real world is decreasing due to the disadvantages of CABG and the improvement in PCI procedures. Thrombolytic therapy has some disadvantages such as low recanalization rate, high risk of reocclusion and bleeding, and short time window. On the other hand, intracoronary interventional therapy may meet the requirements of “early, complete and persistent” patency of coronary arteries at different time points. However, in the emergency PCI, although thrombus aspiration via a catheter or balloon dilation is performed, residual thrombus with heavy or low TIMI (thrombolysis in myocardial infarction) myocardial perfusion grading is still observed in some patients, suggesting disordered microcirculation. Currently, the treatment of microcirculatory disturbance in emergency PCI mainly employed injection of tirofiban, adenosine, thrombolytic agent or other drugs into the local area via a microcatheter in a short time, all of which can significantly reduce the thrombus load and improve TIMI perfusion. Herein, we report that a microcatheter was indwelled in the coronary artery for continuous pumping of low-dose thrombolytic drugs as reperfusion therapy in 12 patients with acute and subacute MI.

## 1. Introduction

Early reperfusion therapy can significantly reduce mortality and improve the cardiac function of patients with acute myocardial infarction (AMI). The reperfusion therapy mainly employs intravenous thrombolysis, percutaneous coronary intervention (PCI), and coronary artery bypass grafting (CABG). Emergency PCI is more effective than intravenous thrombolysis in reducing mortality.^[1,2]^ However, in some cases, recanalization of AMI-related arteries in emergency PCI during and after surgery is inconsistent with the improvement of clinical symptoms, manifested as a small ST-segment rebound amplitude, thrombolysis in myocardial infarction (TIMI) myocardial perfusion grade or low myocardial color. This may be explained as that the recanalization of AMI-related arteries does not mean complete recovery of tissue perfusion. Therefore, it is important to reduce reperfusion time and improve microcirculation perfusion in reperfusion therapy. In clinical practice, a microcatheter was indwelled in the coronary artery for continuous pumping of low-dose thrombolytic drugs in our department. Therefore, the drugs acted selectively on local thrombi, which avoided the side effects of high-dose thrombolytic drugs.

## 2. Subjects and methods

### 2.1. Patients

This study was conducted in accordance with the Declaration of Helsinki, the International Conference on Harmonization, Good Clinical Practice guidelines and all applicable laws and regulations. The study protocol was approved by the Ethics Committee of Nanjing First Hospital, Nanjing Medical University. Twelve patients were recruited from the Department of Cardiology, Nanjing First Hospital of Nanjing Medical University. The inclusion criteria were as follows: patients were diagnosed with acute or subacute ST-segment elevation myocardial infarction (MI) according to the WHO diagnostic criteria; patients received early emergency PCI; there were no contraindications to thrombolysis. Exclusion criteria were as follows: patients were older than 75 years; patients had hemorrhagic disorders; patients were allergic to antiplatelet drugs; there were cerebrovascular accidents, thrombocytopenia, or other complications that were not suitable for emergency treatment, early PCI or thrombolysis. There were 8 males and 4 females with an average age of 62.5 ± 8.6 years. Comorbidities included hypertension in 9 patients, diabetes in 6, hyperlipidemia in 8, and a history of smoking in 6. In these patients, lesions were found in the anterior descending branch (n = 5) and the right coronary artery (n = 7). Coronary angiography showed a very severe initial thrombus load. The thrombus load classification is based on the TIMI thrombus classification (TTG) proposed by Gibson in 2001. The thrombus load is divided into 5 levels: Level 0: no thrombus; Level 1: lumen development is fuzzy; Grade 2: Definite thrombus, but < 1/2 vessel diameter; Grade 3: Thrombus is 1/2–2 times of blood vessel diameter; Grade 4: thrombus ≥ 2 times of blood vessel diameter; Grade 5: thrombus completely blocks the blood vessel. In the case of TTG grades 2 to 5, the thrombus is visible on angiography; TTG ≥ 4 is defined as a high-load thrombus. All the patients had high-load thrombus. After routine thrombus aspiration or balloon dilation, there was still severe thrombus load or reduced TIMI perfusion. Then, coronary artery catheterization was performed for continuous infusion of low-dose thrombolytic drugs for local thrombolysis.

### 2.2. Premedication

All patients were treated with aspirin (300 mg) and Tegretol (180 mg) before operation. Intraoperative heparin was administered at 100 to 120 U/kg. The intraoperative activated clotting time was maintained at 250 to 350 seconds.

### 2.3. Percutaneous transluminal coronary angioplasty (PTCA) and intracoronary microcatheter indwelling

All patients underwent routine coronary angiography through the right radial artery. The thrombus load was severe on coronary angiography and there were indications to interventional treatment. A 6F guiding catheter was used to guide the guidewire to the distal end of the lesion, and a thrombus suction catheter (Export AP) was used for suction. At the same time, tirofiban was injected into the coronary artery. If there was a local thrombus or severe stenosis, non-compliant balloons were used for dilation after above treatment. If there was still severe thrombus load or TIMI perfusion reduction, an extension guidewire (ASHIAI, Extension coronary guidewire [150 cm in length] with a connecting tube at the top end and hollow [15 cm] in the middle; generally about 190 cm in length) was used. On fluoroscopy, the guiding catheter was withdrawn, the guidewire was kept in place, the microcatheter was inserted into the site with severe thrombus load along the guidewire, the radial artery sheath was maintained in place, and the microcatheter was sutured and fixed at the opening of the sheath. Then, a micro-pump was connected, followed by continuous infusion of urokinase at a low dose (2 WU/H) until the end of operation (5 ± 2 minutes).

### 2.4. Post-operative medication and follow-up

All patients received primary and secondary prevention of coronary heart disease. Patients were treated with oral aspirin at 100 mg/d and Tegretol at 180 mg/d, and subcutaneous low molecular heparin (40 mg every 12 hours). Moreover, tirofiban was pumped at 0.15 μg/kg/min for about 36 hours and urokinase was pumped at a low-dose (2 WU/H) via the microcatheter until 72 hours after surgery. During this period, routine blood test, blood biochemical test and coagulation tests were done once daily, and the ST-segment regression, fibrinogen level, bleeding and chest pain were monitored. After 72 hours, coronary angiography was conducted, and selective aortography was employed to observe thrombolysis, TIMI perfusion and thrombus on the surface of microcatheter, and PCI was performed if necessary.

## 3. Results

All 12 patients underwent successful thrombus aspiration and successful microcatheter indwelling. Chest pain was significantly relieved immediately after surgery, and ST-segment regression exceeded 50%. During the thrombolysis period, fibrinogen decreased slightly, and the above outcomes were clinically acceptable. None had bleeding of BARC type 3 or higher. Only 3 patients experienced epistaxis and mucosal bleeding, which resolved after compression with gauzes and symptomatic management. On coronary angiography at 72 hours, the thrombus load significantly reduced or even disappeared, and TIMI perfusion graded as 3. Only 3 patients had local thrombus shadows and visible intimal flaps, and thus 4 stents were implanted at the lesion site. Moreover, a high-pressure injector was used for aortic angiography, and no thrombus adhesion was observed on the surface of microcatheter. All the patients were safely discharged 7 to 10 days after admission. In 2 patients, the procedure and results of aortography were reported (patient 1 was the first case in which this method was used).

**Figure 1.**
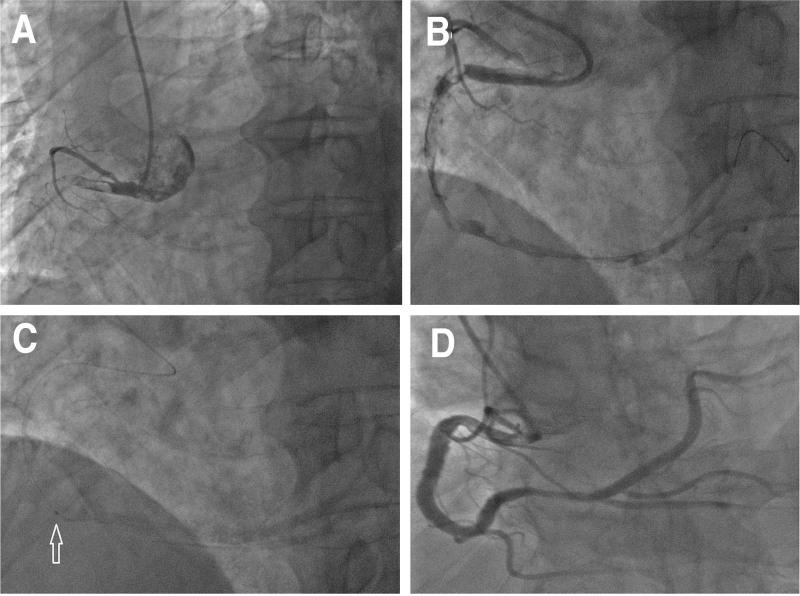
The specific process of Case 1. (A) Selective angiography (left anterior oblique) proximal segment of RCA with a visible large thrombus; (B) Distal segment of RCA on IVUS showed the guidewire in the true lumen and the lumen is filled with thrombus; (C) Retained microcatheter in the middle of RCA (arrow, microcatheter tip); (D) The intracoronary thrombus completely disappeared and the blood was TIMI grade 3 after 48-h intra-RCA urokinase injection (orthocephalic). RCA = right coronary artery, TIMI = thrombolysis in myocardial infarction.

Case 1. A male patient aged 70 years and weighing 68 kg was admitted due to “repeated chest pain for 1 month.” He had a history of hypertension and diabetes, and a DES (3.5 × 18 mm) was implanted in the right coronary artery (RCA) 2 years ago in another hospital. Electrocardiography on admission showed old inferior MI. The blood troponin I level was 0.16 ng/mL, but CK and CK-MB levels were normal. After admission, “dual antiplatelet” drugs at loading doses were administered, and selective coronary angiography (CAG, 2017.6.21) was performed. On coronary angiography, the RCA was completely occluded from the proximal segment, with the forward blood flow at TIMI0 level. The specific process is shown in (Fig. 1A–D)

**Figure 2.**
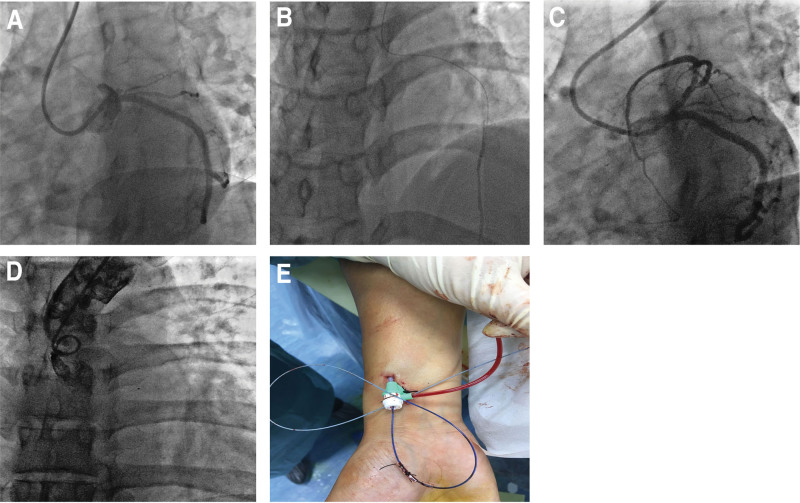
The specific process of Case 2. (A) Posterior thrombotic occlusion of proximal LAD on coronary angiography. (B) High after thrombus aspiration, and thus a microcatheter was remained in place. (C) A significant reduction of thrombus load on repeat coronary angiography 48 h later. (D) Microcatheter surface without unstinting See thrombus attachment on the aortography 48 h later. Fixed microcatheter at puncture site of radial artery without bleeding.

Case 2. A male patient aged 32 years was admitted due to “chest pain for 9 h.” He was diagnosed with extensive anterior MI by myocardial enzyme examination and electrocardiography. After routine aspiration of coronary thrombus, a distal thrombus was still visible and the blood flow was TIMI grade 2. A microcatheter was placed in the anterior descending branch, and low-dose urokinase was administered through the microcatheter for 48 hours. There was no additional bleeding at the puncture site of radial artery. The specific process is shown in Figure 2.

## 4. Discussion

AMI is one of the important clinical critical illnesses. The key to the treatment of AMI is early, rapid and complete recanalization of the lesioned vessels, improvement of reperfusion to the ischemic area, and minimization of myocardial cell necrosis, important for the improvement of short- and long-term prognosis. The commonly used strategies for reperfusion therapy include intravenous thrombolysis, PCI and CABG. However, each strategy has its own advantages and disadvantages. Currently, emergency PCI is recognized as the most effective treatment worldwide. However, in emergency PCI, a heavy thrombus load is often observed, and the blood flow is poor or even no-reflow is present after thrombus aspiration. Coronary microvascular dysfunction caused by microvascular edema, spasm and micro-thromboembolism may lead to severe myocardial ischemia.^[3]^ Generally, simple PCI often fails to remove the thrombus in the coronary artery, and may easily cause the thrombus broken during PCI and its migration to the distal infarct-related artery, resulting in poor microcirculation reperfusion. Poor reperfusion in the myocardial microcirculation can cause arrhythmia, pericardial effusion, congestive heart failure, and left ventricular remodeling in the early stage of MI, and may also significantly increase the readmission and mortality of patients with late-stage heart failure after MI. The inconsistency between recanalization rate of infarct-related vessels and survival rate lies in the fact that the smooth blood flow in the epicardial coronary artery does not mean the complete recovery of microcirculation reperfusion in tissues, the microcirculation is still poor, and the myocardial tissues cannot receive enough blood supply.

Currently, there are several treatments for heavy thrombus load and poor myocardial microcirculation perfusion, one of which is the targeted administration of drugs (such as adenosine and thrombolytic agents) via the coronary arteries. Bulluck et al^[4]^ conducted a meta-analysis of intracoronary adenosine injection for the treatment of acute ST-segment elevation MI and their results showed that intracoronary adenosine injection significantly reduced the incidence of no-reflow compared with administration of adenosine via peripheral veins and significantly decreased the incidence of heart failure. Moreover, It has been already confirmed that the therapeutic effects of coronary intraluminal thrombolysis combined with interventional therapy for acute in-stent thrombosis, left main stem thrombosis, and neonatal acute coronary occlusion.^[5–7]^ Daniela et al^[8]^ reported that, for AMI patients with heavy thrombus load and failure of thrombus aspiration, injection of tenecteplase or alteplase at 1/3 standard dose through a microcatheter could significantly reduce thrombus load and improve myocardial perfusion, with ST-segment regression of more than 50% in 82% of patients and an increase in TIMI grade from baseline 0/1 to ≥ 2 after treatment in 97% of patients, the treatment was safe, and few bleeding complications were observed. The injection of other drugs (such as morphine, verapamil, diltiazem, and scopolamine) into the coronary artery has also been reported to improve coronary blood flow and clinical efficacy.^[9–11]^ The current treatment for coronary thrombosis involves short-term intracoronary injection of drugs, especially thrombolytic drugs. Although short-term intracoronary injection of drugs improves coronary blood flow and clinical efficacy, it is necessary to consider the timeliness of drug-thrombus contact: whether thrombus load continues to decrease, whether microcirculation perfusion continues to improve, whether clinical efficacy continues to increase, and whether the possibility of stent implantation can be reduced. In conjunction with the well-established treatment of acute pulmonary embolism with continuous pumping of thrombolytic drugs from an indwelling thrombolytic catheter in the pulmonary artery,^[12]^ we introduced the continuous pumping of low-dose urokinase from an indwelling microcatheter in the coronary artery to reduce thrombus load and improve microcirculatory perfusion. In this study, 12 patients with acute and subacute ST-segment elevation MI were treated with this strategy. All the patients had a significantly reduced thrombus load on imaging examination 72 hours later, the blood flow of TIMI grade 3 was observed in all the patients, stenting was avoided in 9 patients, and major bleeding was absent in all the patients. The improved efficacy may be explained as follows:

1. The presence of a thrombus in the coronary artery reduces blood flow. In thrombolysis through peripheral veins, the amount of drug reaching the coronary artery is low even when the total amount of drug is equivalent. However, the microcatheter can ensure that thrombolytic drugs act on the thrombus with sufficient concentration for a long time, which avoids the risk of bleeding after short-term medication with high-dose thrombolytic drugs and ensures favorable clinical efficacy. The half-life of general thrombolytic drugs is relatively short, the half-life of commonly used urokinase and alteplase in clinical practice is often shorter than 20 minutes, and drug clearance is extremely quick. The short-term intracoronary injection of thrombolytic drugs cannot guarantee complete lysis of the thrombus, while long-term continuous infusion of thrombolytic drugs can lyse the thrombus as much as possible, which improves microcirculation perfusion.
2. In this study, thrombus aspiration combined with thrombolysis was employed. Mechanical aspiration can cause the exposure of inner thrombus, which increases total area of action of thrombolytic drugs, thereby enhancing the thrombolytic effect of drugs. Therefore, intracoronary microcatheter-targeted thrombolysis can dissolve thrombi in large blood vessels outside the heart and improve myocardial microvascular perfusion. Moreover, the dose of drugs can be adjusted according to the disease condition, which also reduces risk of bleeding.

Under the medication of normal oral “double anti-platelet drugs,” low molecular weight heparin and GPI/low-dose urokinase, long-term indwelling of microcatheter in the coronary arteries is safe. The main concern of intracoronary catheter indwelling is the formation of extramural thrombus on the catheter. In this study, 1 patient had indwelling of microcatheter in the middle RCA for up to 6 days, but no thrombus was found outside the catheter on follow-up coronary angiography and direct observation. Especially, coronary angiography confirmed that there was no thrombus on the outer surface of microcatheter near the right coronary artery. The shear force of aortic blood flow was significantly larger than the blood flow of coronary artery, and theoretically, it is more likely to form an extramural thrombus in the intracoronary catheter than in the intravascular catheter. The safety of intracoronary microcatheter indwelling has also been supported by clinical treatments of other thrombotic diseases. In case of pulmonary embolism, thrombolysis with low-dose urokinase is performed via a catheter indwelled in the pulmonary artery, and extramural thrombus formation outside the catheter is rare in both right ventricle and venous segment, which strongly supports the safety of intracoronary catheter indwelling.

There were still limitations in this study. This was an observational study. Only 12 patients were included in this study, and the sample size was small. The patients included in this study were relatively young and the risk of bleeding was low in these patients. More randomized controlled studies with large sample sizes are needed to confirm the efficacy of this strategy and risk of bleeding in AMI patients receiving this treatment.

## Acknowledgments

We thank all the participants for their participation in the present study.

## Author contributions

**Conceptualization:** Yuzhu Chen, Yuan Liu.

**Formal analysis:** Yuzhu Chen, Yuan Liu.

**Investigation:** Xiaohui Li.

**Methodology:** Xiaohui Li.

**Supervision:** Guozhen Jin.

**Visualization:** Yuan Liu.

**Writing – original draft:** Yuzhu Chen.

**Writing – review & editing:** Guozhen Jin.
